# Cell-wide arrangement of Golgi/RE units depends on the microtubule organization

**DOI:** 10.1247/csf.24055

**Published:** 2024-10-03

**Authors:** Tatsuya Tago, Syara Fujii, Shogo Sasaki, Maki Shirae-Kurabayashi, Naoaki Sakamoto, Takashi Yamamoto, Makoto Maeda, Tatsuya Ueki, Takunori Satoh, Akiko K. Satoh

**Affiliations:** 1 Program of Life and Environmental Science, Graduate School of Integral Science for Life, Hiroshima University, Higashi-Hiroshima, Hiroshima 739-8521, Japan; 2 Sugashima Marine Biological Laboratory, Graduate School of Science, Nagoya University, Toba, Mie 517-0004, Japan; 3 Program of Biomedical Science, Graduate School of Integrated Sciences for Life, Hiroshima University, Higashi-Hiroshima, Hiroshima 739-8521, Japan; 4 Natural Science Center for Basic Research and Development, Hiroshima University, Higashi-Hiroshima, Hiroshima 739-8526, Japan; 5 Program of Basic Biology, Graduate School of Integrated Sciences for Life, Hiroshima University, Higashi-Hiroshima, Hiroshima 739-8521, Japan

**Keywords:** Golgi stack, recycling endosome, Golgi-ribbon, microtubule, cilium, sea urchin, ascidian

## Abstract

We have previously shown that Golgi stacks and recycling endosomes (REs) exist as Golgi/RE units in sea urchin embryos. In this study, we showed that Golgi/RE units were scattered throughout the cytoplasm at early developmental stages but gathered to form a “Golgi ring” surrounding the centric REs at the blastula stage. This change in the cell-wide arrangement of Golgi/RE units coincided with a dramatic change in microtubule organization from a randomly oriented cortical pattern to radial arrays under the apical plasma membrane. A single gigantic Golgi apparatus surrounding centric RE is clearly associated with the center of the radial microtubule arrays. Furthermore, we found that in some animal species belonging to different clades, Golgi stacks lack lateral connections but are likely centralized by microtubule motors. These results suggest that Golgi centralization depends on the organization of the microtubule array in addition to the lateral linking between Golgi stacks.

## Introduction

The Golgi apparatus is the central organelle in the secretory pathway, and is responsible for the modification and sorting of lipids, membranes, and secreted proteins to various cellular destinations (Klumperman, 2011; Nakano, 2022; Papanikou and Glick, 2014). The basic structural unit of the Golgi apparatus is the Golgi stack, which consists of several flattened cisternae, tubules, and vesicles. Recycling endosomes (REs) are perinuclear compartments through which endocytosed materials are trafficked before being recycled back to the plasma membrane and are well defined in mammalian cells (Mayor *et al.*, 1993; Taguchi, 2013; Yamashiro and Maxfield, 1987). Although the Golgi and the RE function mainly in the exocytic and endocytic pathways, respectively, the later work reveals that they also function in the other pathways (Ang *et al.*, 2004; Lock and Stow, 2005; Satoh *et al.*, 2005). We previously reported that the *trans*-side of most Golgi stacks is accompanied by REs in *Drosophila*, microtubule-disrupted HeLa cells, and sea urchin embryos (Fujii *et al.*, 2020a; 2020c). Therefore, the Golgi stacks and REs can exist as Golgi/RE units. Furthermore, REs can exist in free form, and Golgi-associated REs (GA-REs) and free REs are interchangeable.

In plant cells, as well as in *Caenorhabditis elegans* and *Drosophila*, Golgi stacks are known to be dispersed throughout the cytoplasm rather than connected to each other (Gosavi and Gleeson, 2017; Wei and Seemann, 2017). In contrast, in mammalian cells, most Golgi stacks are connected by lateral links, forming a Golgi ribbon that is positioned near the centrosomes via the activity of dynein, a microtubule-based motor (Harada *et al.*, 1998; Saraste and Prydz, 2019; Yadav and Linstedt, 2011). Perinuclear positioning of REs is also known to be microtubule-dependent (Sakai *et al.*, 1991; Ullrich *et al.*, 1996). A remarkable arrangement of Golgi stacks and REs has been observed in COS1 cells: REs form a clump around the centrosome and are encircled by a “Golgi-ring,” with the *trans*-side of the Golgi facing the REs (Misaki *et al.*, 2007; 2010). However, the relationship between centralized Golgi stacks and the REs has not been thoroughly investigated in other mammalian cells and animals.

In this study, we examined the cell-wide arrangement of Golgi/RE units in ascidian, sea urchin embryos and other animals. Our results shed light on the mechanism by which this arrangement is formed.

## Result and Discussion

### A RE-centered U-shaped unified Golgi apparatus in embryonic sea urchin cells

A developmental change in the cell-wide arrangement of Golgi stacks in sea urchin embryos was reported two decades ago (Terasaki, 2000) and replicated in a recent study (Benvenuto *et al.*, 2024). Until the ninth cleavage, Golgi stacks are scattered throughout the cytoplasm, similar to those in plants and fruit flies. However, after the ninth cleavage, all Golgi stacks within the cells coalesce to form a single apical Golgi apparatus. We have previously shown that nocodazole treatment of the late blastula of sea urchin embryos disperses Golgi stacks that are accompanied by REs on their *trans*-sides, forming Golgi/RE units (Fujii *et al.*, 2020c). In this study, we aimed to clarify the distribution of Golgi/RE units in drug-free sea urchin embryos after the ninth cleavage. Due to the difficulty in the cross-reactivity of antibodies, we used mRNA injection to visualize the Golgi stack and RE, similar to a previous study (Fujii *et al.*, 2020c).

**Fig. 1:**
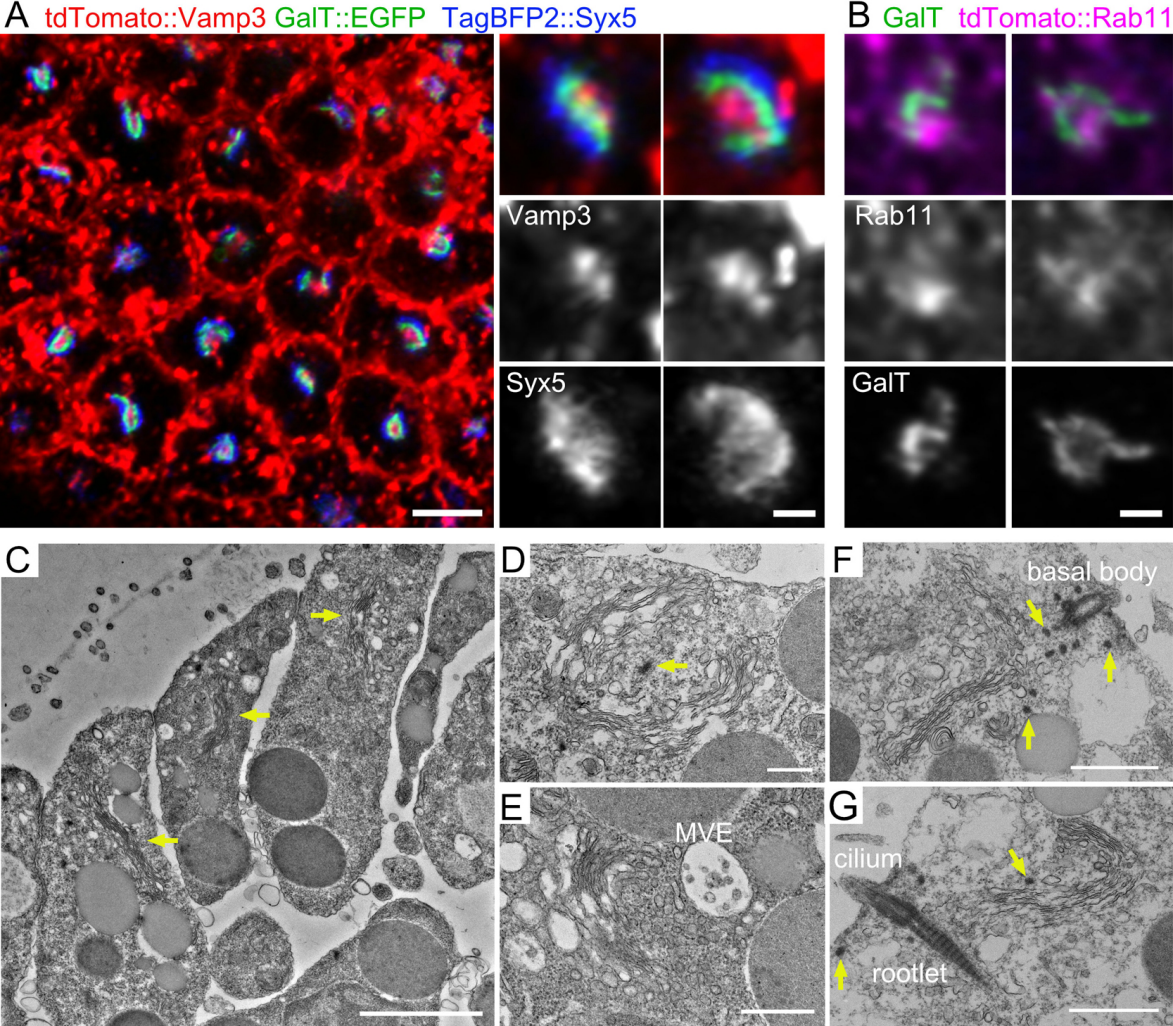
Cell-wide distribution of Golgi/REs in late-blastula-stage sea urchin embryos (A) *Cis*-Golgi cisternae, *trans*-Golgi cisternae, and RE at the late blastula stage of the sea urchin *Hemicentrotus pulcherrimus* visualized using the TagBFP2::Syx5 (blue), GalT::EGFP (green), and tdTomato::Vamp3 (red) after mRNA injections into fertilized oocytes. (B) *Trans*-Golgi cisternae and RE at the late blastula stage of the sea urchin *H. pulcherrimus* visualized using GalT::EGFP (green) and tdTomato::Rab11 (magenta) after mRNA injections into fertilized oocytes. (C–G) Electron micrograph of cells at the late blastula stage of the sea urchin *Heliocidaris crassispina*. Arrows in C indicate gigantic Golgi apparatus. D–G are higher magnification images of the Golgi apparatus in other sections. Arrows indicate electron-dense materials. Scale bars: 5 μm (A left), 1 μm (A right, B), 2 μm (C), 500 nm (D, E), and 1 μm (F, G).

We microinjected mRNAs encoding the *cis*-Golgi marker TagBFP2::Syx5 (Satoh *et al.*, 2016), the *trans*-Golgi marker GalT::EGFP, and the RE marker tdTomato::Vamp3 (Mallard *et al.*, 2002) into fertilized eggs of the sea urchin *Hemicentrotus pulcherrimus* and then incubated them for more than 20 h at 11°C, letting the embryos to develop beyond the blastula stage (Takemoto *et al.*, 2016). As previously reported, each cell contained a single apical Golgi apparatus (Fig. 1A). We found that this Golgi apparatus had a U-shaped profile, and the *cis*- and *trans*-cisternae were apparently continuous and formed a single sheet within this single Golgi apparatus. The *cis*-Golgi cisternae were localized on the outside of the *trans*-Golgi cisternae, whereas the RE was localized in the center of the Golgi apparatus (Fig. 1A). In the blastula stage embryos injected with mRNAs for GalT::EGFP and another RE marker, tdTomato::Rab11, the *trans*-Golgi cisternae also surrounded a clump of Rab11-positive RE (Fig. 1B). This Golgi/RE arrangement was similar to that observed in COS1 cells. However, in sea urchin embryonic cells, this arrangement was U-shaped, whereas in COS1 cells, this was ring-shaped (Misaki *et al.*, 2007; 2010).

Transmission electron microscopy (TEM) revealed a single giant Golgi apparatus in each cell (Fig. 1C, arrows). Within the Golgi apparatus, a couple of continuous cisternae were stacked and elongated into a U-shape (Fig. 1C–G). In the center of the U-shape, a multivesicular endosome accompanied by many vesicles was observed (Fig. 1E). Notably, near the Golgi apparatus, a basal body (Fig. 1F) or a cilium was observed, whose rootlet extended to the interior of the cell (Fig. 1G) (Vlijm *et al.*, 2018; Yang *et al.*, 2002). Near a cilium and the Golgi apparatus, several dots with similar electron density to the cilium were also observed (Fig. 1D, F, G arrows). These are likely to be centriolar satellites (Kubo *et al.*, 1999; Renaud and Bidère, 2021).

### Arrangement of the RE-centered Golgi ring is common in deuterostomes

**Fig. 2:**
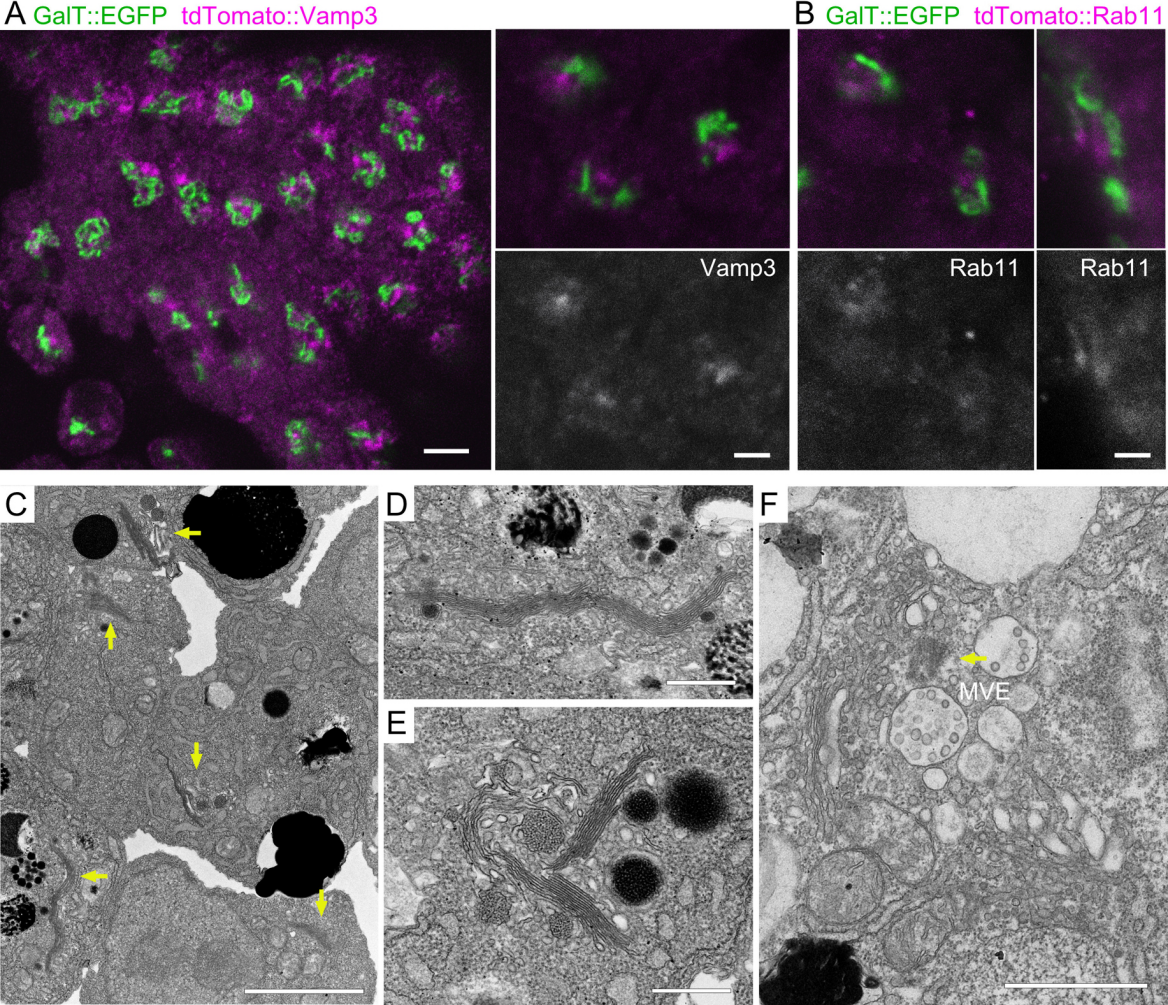
Cell-wide distribution of Golgi/REs in *Ciona robusta* embryos at tailbud stage (A) *Trans*-Golgi cisternae and RE of tailbud-staged ascidian, visualized using GalT::EGFP (green) and tdTomato::Vamp3 (magenta) after mRNA injections into fertilized oocytes. (B) *Trans*-Golgi cisternae and RE of tailbud stage ascidian, visualized using GalT::EGFP (green) and tdTomato::Rab11 (magenta) after mRNA injections into fertilized oocytes. (C–F) Electron micrograph of cells in of tailbud stage ascidian. Arrows in C indicate gigantic Golgi apparatus. D–F are higher magnification images of the Golgi apparatus in other sections. The arrow indicates the centriole. Scale bars: 5 μm (A left), 2 μm (A right, B), 2 μm (C), and 500 nm (D–F).

To investigate the generality of this Golgi/RE arrangement, we examined the Golgi and RE distribution in embryos of the ascidian *Ciona robusta* (*C. intestinalis* type A). Since a developmental rearrangement of the distribution of Golgi stacks has also been reported in the ascidian embryo, we used tailbud stage embryos, in which a cell should have a single Golgi apparatus. We microinjected mRNAs encoding the *trans*-Golgi marker GalT::EGFP and the RE markers tdTomato::Vamp3 or tdTomato::Rab11 into fertilized ascidian eggs and incubated them for 14 h at 17°C and fixed them. We observed a clear configuration of an RE clump surrounded by the Golgi apparatus in each cell, regardless of whether the REs were visualized with tdTomato::Vamp3 or tdTomato::Rab11 (Fig. 2A, B). However, the *trans*-Golgi cisternae visualized by GalT::EGFP did not form a continuous smooth sheet but rather appeared lumpy. Electron micrographs revealed a single unified Golgi apparatus in each cell (Fig. 2C–F). These Golgi apparatuses were elongated or folded into complex structures (Fig. 2D, E). In some ultrathin sections, endosomes were clustered near the basal body and Golgi stacks surrounded the endosomes, with the *trans*-side facing the clustered endosomes (Fig. 2F). These results indicate that the RE-centered unified Golgi apparatus is a common configuration of the Golgi apparatus and the RE in deuterostomes.

### Radial microtubule array from centrosome centralizes Golgi apparatus and RE

The cell-wide distribution of Golgi stacks in sea urchin embryos changes dramatically after the ninth cleavage from scattered Golgi stacks to a centralized Golgi apparatus. At about the same time, sea urchin embryos develop a ciliated epithelium. Since cilia or basal bodies were found in the center of the Golgi apparatus and endosomes in electron micrographs, we hypothesized that the dramatic change in microtubule organization at this stage induces both the centralization of Golgi stacks and the elongation of cilia.

**Fig. 3:**
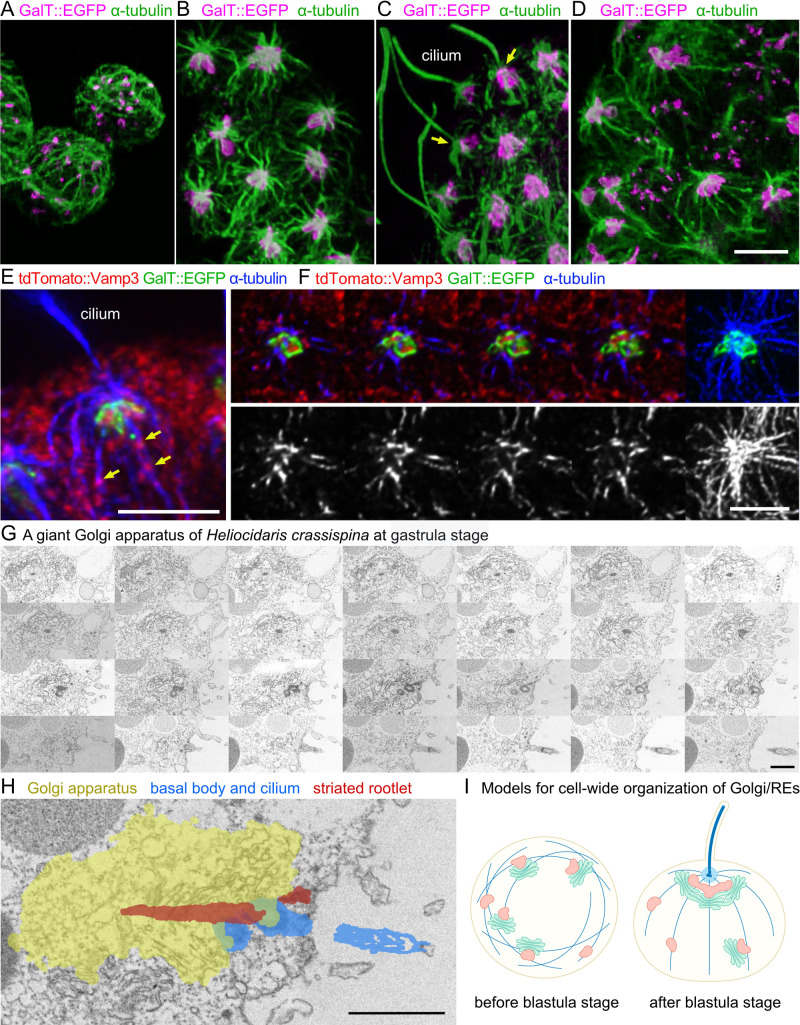
Formation of a giant Golgi apparatus under the center of the microtubule organizing center at late blastula stage (A–D) Projection images of 43 (A), 31 (B) or 34 (C, D) sections with 0.3-μm interval in embryo cells of the sea urchin *H. pulcherrimu*. *Trans*-Golgi cisternae and microtubules were visualized using GalT::EGFP (magenta) after the mRNA was injected into fertilized oocytes. The samples were immunostained with anti-α-tubulin antibody (green). (A) Preblastula, (B, C) late blastula, and (D) intermediate stages. Arrows indicate MTOC. (E, F) *Trans*-Golgi cisternae, RE, and microtubules at the late blastula stage of the sea urchin *H. pulcherrimus* visualized using GalT::EGFP (green) and tdTomato::Vamp3 (red) after their mRNA was injected into fertilized oocytes. The samples were immunostained with anti-α-tubulin antibody (blue) at the late blastula stage. Arrows indicate REs on microtubules. Left and right are the projection images of 15 and 26 z-sections at 0.3-μm intervals, respectively. (G) 30-nm thick serial sections of the Golgi apparatus in a cell of the gastrula-stage sea urchin *H. crassispina*, observed by scanning electron microscopy. (H) Electron micrograph showing projections of the Golgi apparatus (yellow), cilia(blue), basal body (blue), and striated rootlet (red) made from the serial sections in (G). (I) Models for the cell-wide arrangement of Golgi/RE units in a cell at the gastrula stage of the sea urchin. Golgi stacks in green, REs in Red, microtubules and basal body in blue. Scale bars: 5 μm (A–F), and 1 μm (G, H).

To test this hypothesis, we examined the organization of the Golgi apparatus and microtubules before and after the ninth cleavage. Before the ninth cleavage, the microtubules were not centralized and randomly oriented in a cage-like pattern under the plasma membrane, and Golgi stacks were scattered throughout the cytoplasm (Fig. 3A). This pattern of microtubule organization is similar to that observed in *Drosophila* S2 cells (Fujii *et al.*, 2020b; Rogers *et al.*, 2008; Wegel *et al.*, 2016). The association between microtubules and Golgi stacks was not evident at this stage. In contrast, after the ninth cleavage, radial microtubule arrays formed beneath the apical plasma membrane, and a single gigantic Golgi apparatus was clearly associated with the center of the microtubule arrays (Fig. 3B and Movie 1). The Golgi apparatus was positioned just below the microtubule-organizing center (MTOC), which was presumably the basal body. At a slightly later stage, the ciliary microtubules extended from this region (Fig. 3C). Interestingly, the embryos contained two types of cells, one with randomly oriented microtubules and the other with radial microtubules. The former had scattered Golgi stacks, and the latter had a gigantic Golgi apparatus (Fig. 3D). To clarify the localization of RE and Golgi apparatus against a MTOC, embryos injected with both GalT::EGFP and tdTomato::Vamp3 were immunostained with anti-α-tubulin. Notably, RE clumps were localized in the center of the Golgi apparatus just below the MTOC (Fig. 3E, F and Movies 2, 3). In addition, some RE puncta were localized to microtubules straying from the MTOC (Fig. 3E, arrows).

**Fig. 4:**
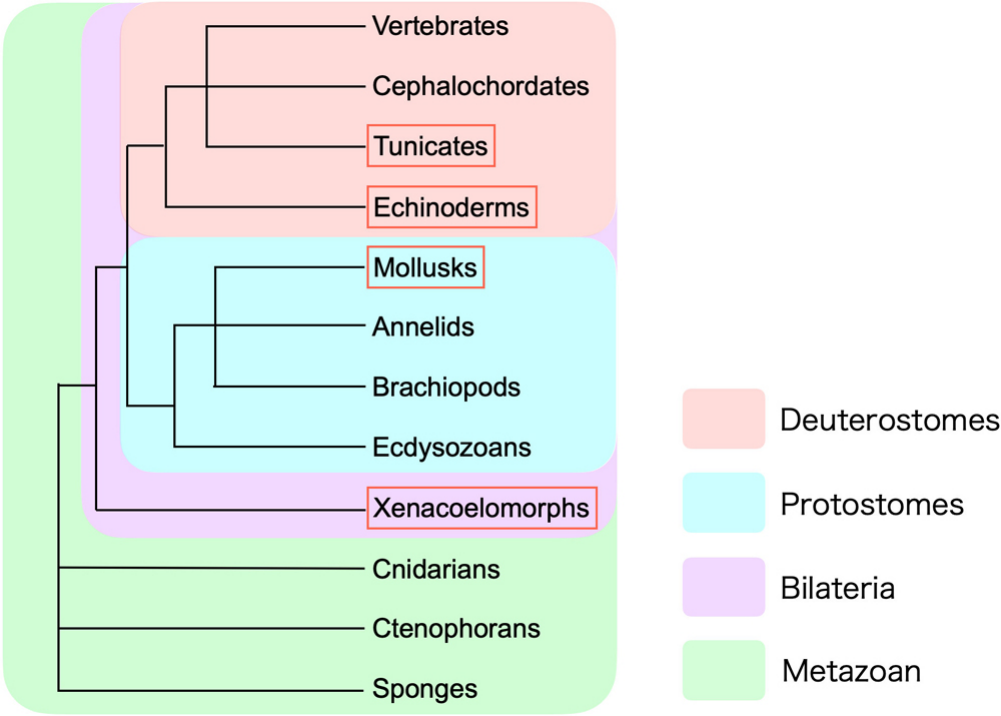
Schematic of animal evolution in metazoans The schematic represents the phylogenetic tree of metazoans, adapted from Adoutte *et al.* (Adoutte *et al.*, 2000) and Ueki *et al.* (Ueki *et al.*, 2019). The phyla to which the animals used in the experiments belong are highlighted with red rectangles. Sea urchins *H. pulcherrimus* and *H. crassispina* belong to echinoderms, the ascidian *C. robusta* belongs to tunicates, the marine acoel worm *P. naikaiensis* belongs to xenacoelomorphs, and the mollusk *A. esculentum* belongs to mollusks.

To clarify the localization of the cilia, basal bodies, striated rootlets, and Golgi apparatus, we examined serial thin sections by scanning electron microscopy (SEM). The basal body was localized near the plasma membrane at the base of the cilium, and the striated rootlet extended from the bottom of the basal body to the center of the U-shaped Golgi apparatus in some cells (Fig. 1D, 3G, H and Movie 4). In other cells, the striated rootlet was localized close to, but not inside the Golgi apparatus (Fig. 1G and Movie 5). Tachi *et al.* reported this close localization of the Golgi apparatus and the striated rootlet in rat uterine epithelial cells and discussed the possibility of a function for the striated rootlet in secretion (Tachi *et al.*, 1974). Several electrodense foci, presumably centriolar satellites, were observed near the base of the cilia. These results suggest that the organization of radial microtubule arrays in the cytoplasm accompanied by ciliogenesis centralizes Golgi/RE units in sea urchin embryos (Fig. 3I). In mammalian cells, recycling endosomes are known to localize at the base of the cilium and Rab11 and Rab8 are known to function in ciliogenesis (Knödler *et al.*, 2010; Zhao *et al.*, 2023). Thus, our results suggest that the function of recycling endosomes in ciliogenesis is conserved in deuterostomes (Fig. 4).

### Clustering of Golgi stacks and Golgi-ribbon formation are independent processes

Golgi clustering and ribbon formation are believed to be restricted to vertebrates and deuterostomes. However, a recent study showed that the ribbon-like architecture of the Golgi apparatus is common in cells of several metazoan taxa (Benvenuto *et al.*, 2024). Their data suggest that the ribbon-like Golgi apparatus evolved in the common ancestor of cnidarians and bilaterians and was secondarily lost in xenacoelomorphs, arthropods, and nematodes. Intriguingly, they showed that in the marine worm *Symsagittifera roscoffensis* (a xenacoelomorph), some secretory cells had closely adjacent but clearly distinct Golgi stacks (Fig. 1B and S1B in Benvenuto *et al.*, 2024). In these electron micrographs, endosome-like structures are localized at the center of these Golgi stacks, which appear to have their *trans*-side facing the central endosome-like structures. The endoplasmic reticulum surrounds these clustered Golgi stacks, suggesting that the *cis*-side of the Golgi stacks faces outward. Although the Golgi stacks are not laterally connected, the cell-wide arrangement of Golgi and endosomes in *S. roscoffensis* is similar to that of Golgi/RE units in ascidian/sea urchin embryos and COS1 cells.

Benvenuto *et al.* proposed that the evolution of the binding between the Golgi reassembly and stacking protein (GRASP) and the Golgin tethers have driven the link between Golgi stacks, resulting in a ribbon-like configuration (Benvenuto *et al.*, 2024). Alphafold2 prediction successfully revealed GRASP-Golgin binding in cnidarians and most bilaterians but failed to detect their interaction in *Drosophila* (anthropod), *C. elegans* (nematode), and *Hofstenia miamia* (xenacoelomorph). Their results in xenacoelomorphs showed that Golgi stacks are centralized but not laterally connected, and GRASP-Golgin binding is not maintained, suggesting that Golgi stack centralization and ribbon formation can be considered independently.

**Fig. 5:**
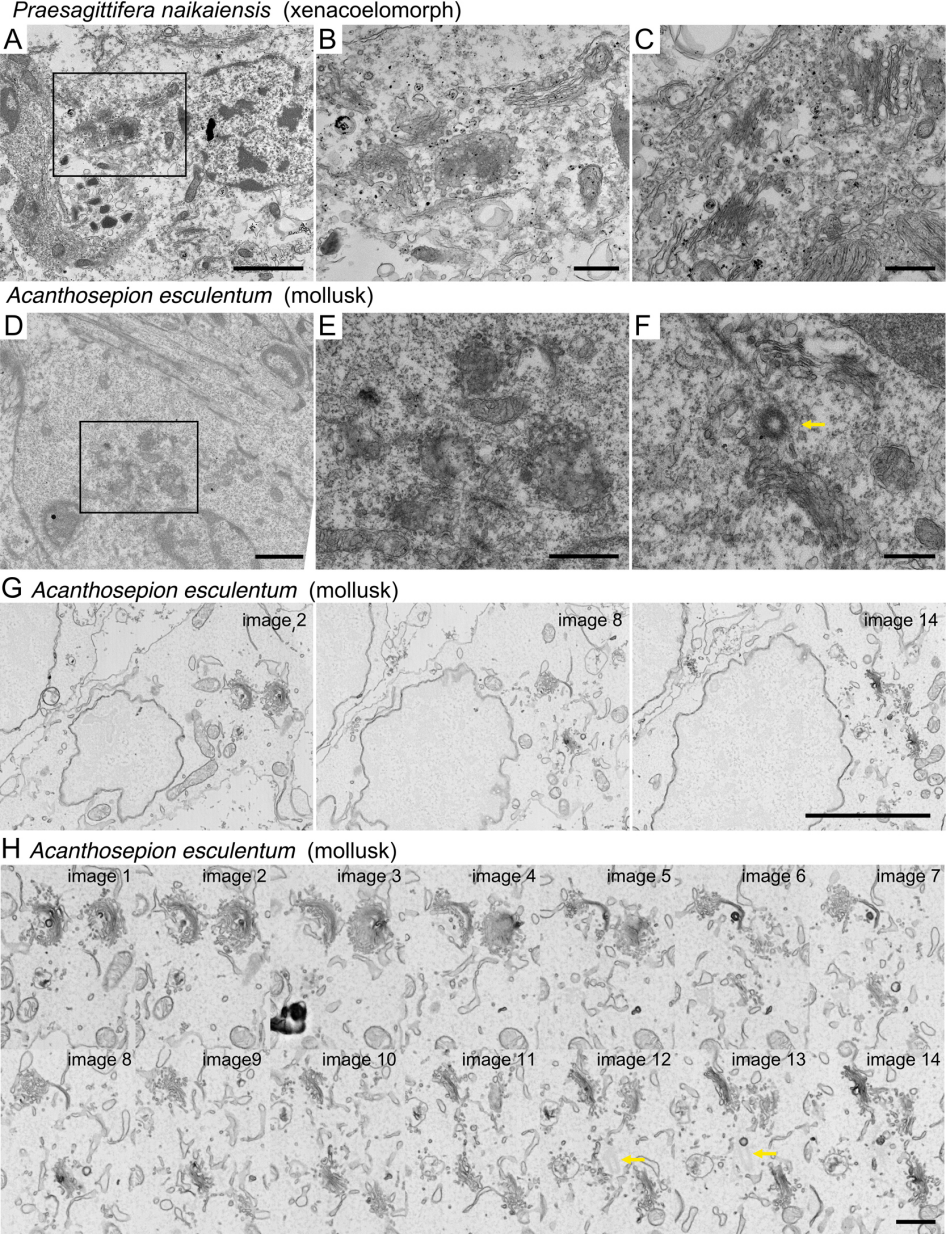
Golgi stacks clustered near centrosomes in *Praesagittifera naikaiensis* and *Acanthosepion esculentum* (A–C) An electron micrograph of a cell in the xenacoelomorph *P. naikaiensis*. B is magnified images of clustered Golgi stacks in a square in A. C is the clustered Golgi stacks in another specimen. (D–F) Electron micrograph of a cell in the embryo of the mollusk *A. esculentum*. E is magnified images of clustered Golgi stacks in a square in D. F is the clustered Golgi stacks in another specimen. The arrow indicates the centriole. (G, H) 90-nm thick serial sections of clustered Golgi stacks in an embryonic cell in the mollusk *A. esculentum* observed by scanning electron microscopy. H is magnified images of G. The arrows indicate the centriole. Scale bars: 2 μm (A), 500 nm (B, C), 2 μm (D), 1 μm (E), 500 nm (F), 5 μm (G), and 1 μm (H).

We were able to replicate their observation of centralized but unconnected Golgi stacks in another xenacoelomorph, *Praesagittifera naikaiensis* (Fig. 5A–C). Intriguingly, we found similar clustered but unconnected Golgi stacks in a mollusk, *Acanthosepion esculentum*, in a single ultrathin section using conventional transmission electron microscopy (Fig. 5D–F). Centrioles were occasionally observed near the Golgi stacks (Fig. 5F, arrow). To confirm the centralization but the lack of lateral connection in *A. esculentum*, we performed SEM observations of serial sections at 90 nm. We observed that multiple cup-shaped Golgi stacks without lateral connections were clustered around the centriole (Fig. 5G, H and Movie 6). These results suggest that, at least in a mollusk, *A. esculentum*, Golgi stacks gather around the centrosomes, but do not form a connected Golgi ribbon, probably because of the absence of the Grasp-mediated lateral linking mechanism.

### Conclusion and future perspective

In most vertebrate cells, Golgi stacks are laterally linked to form a Golgi ribbon and are positioned near the centrosome by the action of the dynein microtubule motor (Harada *et al.*, 1998; Saraste and Prydz, 2019; Yadav and Linstedt, 2011). Benvenuto *et al.* examined the distribution of Golgi stacks and the structure of GRASP across various animal taxa, and they proposed that Golgi centralization is mediated by GRASP-dependent lateral links (Benvenuto *et al.*, 2024). In this study, in ascidian and sea urchin embryos at the blastula stage, we investigated the cell-wide arrangement of Golgi/RE units and found that Golgi stacks form a “Golgi-ring” surrounding the centric REs. This configuration likely depends on the radial array of microtubules from the centrosomes. In addition, our results suggest that in some animals lacking lateral connections, Golgi stacks can be centralized by microtubule motors. Therefore, centralization and ribbon formation in Golgi stacks should be considered separately. The presence of Golgi/RE units and the concentration of RE in the centrosomes may play a role in the centralization of Golgi stacks. Further studies are needed to elucidate the complete mechanism underlying the cell-wide arrangement of Golgi/RE units.

## Materials and Methods

### Animals and embryos

Adult sea urchins (*H. pulcherrimus*) were collected from the Seto Inland Sea and Tateyama Bay. Eggs and sperm were obtained by coelomic injection of 0.55 M KCl. Fertilized eggs were cultured in filtered seawater at 11°C.

Adult ascidians (*C. robusta*) collected from Maizuru Bay (Kyoto) were obtained from the National BioResource Project (NBRP), MEXT, Japan. Adults were maintained under constant light to induce oocyte maturation. Eggs and sperm were obtained surgically from the gonoduct. The marine acoel worm *P. naikaiensis* (Hikosaka-Katayama *et al.*, 2020) and embryos of a mollusk *A. esculentum* were collected at seashores of the Seto Inland Sea, Japan.

### Construction and injection of mRNA

For in vitro transcription of mRNA, the template DNA were amplified from pMT-2xtagBFP2-Syx5, pMT-GalT-EGFP-T2A-tdTomato-Rab11, or pMT-GalT-EGFP-T2A-tdTomato-Vamp3 (Fujii *et al.*, 2020b), using KOD plus neo (TOYOBO, Osaka, Japan) and the following primers: T7-MT-F (5'-TAATACGACTCACTATAGGGtcagcagcaaaatcaagtgaatcat-3') and SV40-pA (5'-ttttttttttttttttttttttttttttttcactgcattctagttgtggtttgt-3'). From 1 μg of template DNA, capped and poly-A tailed mRNA were transcribed by using HiScribe T7 ARCA mRNA Kit with Tailing (NEB, Ipswich, UK), then purified using Zymo RNA Clean & Concentrator-25 (Zymo Research, Irvine, CA, USA) and eluted in water. The mRNA was mixed with glycerol at a final concentration of 40% and then microinjected at a final concentration of 5 ng/μL as previously described (Rast, 2000).

### Immunohistochemistry

Notably, PLP (10 mM periodate, 75 mM lysine, 30 mM phosphate buffer, and 4% paraformaldehyde) was used as a fixative. Mouse monoclonal anti-α-tubulin (1:50 concentrated supernatant; Developmental Studies Hybridoma Bank, Iowa City, IA, USA) and Alexa Fluor 488 conjugated anti-mouse antibodies (1:300; Life Technologies, Carlsbad, CA, USA) were used.

### Image acquisition and analysis

Images of the samples were recorded using a FV3000 confocal microscope (UPLXAPO60XO 1.30 NA and UPlanSApo 60 × S2 1.42 NA objective lens; Olympus, Tokyo, Japan). To minimize bleed-through, each signal in double- or triple-stained samples was sequentially imaged. Images were processed according to the guidelines for proper digital image handling using ImageJ and/or Affinity Photo (Serif Europe Ltd., West Bridgford, Nottinghamshire, UK) (Schindelin *et al.*, 2012).

### Transmission electron microscopy observation

Animals were fixed in 2% paraformaldehyde, 2% glutaraldehyde, 0.1 M phosphate buffer saline with 250 mM NaCl pH 7.4 for >1 day. Samples were then post-fixed (2% OsO_4_, 0.1 M cacodylate buffer, pH 7.4), stained with 2% uranyl acetate, dehydrated through a graded series of ethanol, and embedded in epoxy resin (Quetol-812, Nisshin EM Co. Ltd, Tokyo, Japan). The ultrathin sections were stained with lead hydroxide. The samples were observed under JEM-1400 and JEM-1400Plus electron microscopes (JEOL, Tokyo, Japan), and images were obtained using a CCD camera system (JEOL).

### Serial section scanning electron microscopy observation

Embryos of *A. esculentum* were fixed in 2% paraformaldehyde, 2% glutaraldehyde, 0.1 M PBS with 250 mM NaCl pH 7.4, and incubated for >1 day. The samples were then post-fixed (2% OsO_4_-KFeCN, mixed with 3% KFeCN/2x PBS and an equal volume of 4% osmium tetroxide); stained with a filtered thiocarbohydrazide solution, 2% OsO_4_, lead aspartate solution, and 2% uranyl acetate; dehydrated through a graded series of cold ethanol and acetone; and embedded in epoxy resin (Quetol-812, Nisshin EM Co. Ltd). The samples prepared for TEM were used to observe sea urchins *H. crassispina* and *P. naikaiensis*.

Serial-section scanning electron microscopy was performed using a high-resolution field-emission scanning electron microscope and a back-scattered electron detector. Serial ultrathin sections (thickness of *A. esculentum* and *P. naikaiensis*: 90 nm and *H. crassispina*: 30 nm.) were cut using a diamond knife (Syntek, SYM2035WT Ultra, Tokyo, Japan) and placed on hydrophilized silicon wafers. The sections were then stained with lead staining solution (Sigma-Aldrich, St. Louis, MO, USA) for 3 min at room temperature. Serial sections were observed using a focused ion beam and scanning electron microscope (HeliosG4 UC, Thermo Fisher Scientific, Waltham, MA, USA) equipped with Maps 3.19, a circular backscattered electron detector (CBS), and an in-lens backscattered electron detector (TLD). The observations were conducted at an accelerating voltage of 3 kV, an irradiation current of 0.4 nA, and a working distance of 4.0 mm using TLD for *A. esculentum* and 3.5 mm using CBS for *H. crassispina*.

## Author Contributions

TS and AS designed the study; TT, SF, and SS performed most of the laboratory experiments and analyzed the data; MS-K and NS performed the experiments and TY supervised the injection of mRNA into the ascidian and sea urchin embryos. MM operated the Helios G4 instrument for volume EM. TU prepared the staged animals. TS and AS supervised all aspects of the project. TS and AS wrote the manuscript, with input and final approval from TT, FS, SS, MS-K, NS, TY, MM, and TU.

## Conflict of Interest

All authors have read and approved the manuscript and declare no financial conflicts of interest.

## Funding

This work was supported by grants from the Japan Society for the Promotion of Science (JSPS) (KAKENHI grant no. 22H02617) to A.K.S. and (KAKENHI grant no. 19K06566) to T.S.; the Japan Science and Technology Agency (JST) (PRESTO grant no. 25-J-J4215, and CREST grant no. JPMJCR22E2) to A.K.S. and (SPRING grant no. JPMJSP2132) to T.T.; Core Research for Organelle Diseases funding from Hiroshima University; Takeda Science Foundation, and Ohsumi Frontier Science Foundation to A.K.S. (Grant Number JP22H04926).

## Abbreviations

REs: recycling endosome
GA-RE: Golgi-associated RE
TEM: transmission electron microscope
SEM: scanning electron microscope
MTOC: microtubule-organizing center
GRASP: Golgi reassembly and stacking protein

## Supplementary Material

Movie 1. A giant Golgi apparatus under the microtubule array. Projection images made from 31 sections with 0.3-μm intervals in the embryo cells of the sea urchin *Hemicentrotus pulcherrimu*. *Trans*-Golgi cisternae and microtubules were visualized using GalT::EGFP (magenta) after the mRNA was injected into fertilized oocytes. The samples were immunostained with anti-α-tubulin antibody (green) at late blastula stage. Scale bars: 5 μm.

Movie 2. Side view of a giant Golgi apparatus and RE under the microtubule array and cilium. A movie made from 55 sections with 0.3-μm intervals in the embryo cells of the sea urchin *H. pulcherrimu*. *Trans*-Golgi cisternae, REs, and microtubules were visualized using GalT::EGFP (green) and tdTomato::Vamp3 (red) after injecting their mRNA into fertilized oocytes. The samples were immunostained with anti-α-tubulin antibody (blue) at the late blastula stage. Scale bars: 5 μm.

Movie 3. A giant Golgi apparatus and RE under the microtubule array and cilium. A movie made from 23 sections with 0.3-μm intervals in the embryo cells of the sea urchin *H. pulcherrimu*. *Trans*-Golgi cisternae, REs, and microtubules were visualized using GalT::EGFP (green) and tdTomato::Vamp3 (red) after injecting their mRNA into fertilized oocytes. The samples were immunostained with anti-α-tubulin antibody (blue) at the late blastula stage. Scale bars: 5 μm.

Movie 4. Rootlet of a cilium wrapped by a giant Golgi apparatus in *Heliocidaris crassispina*. A movie was made from 30 nm-thick 28 sections of a giant Golgi apparatus (yellow), basal body (blue), cilium (blue), and its rootlet (red) in the cells of the sea urchin embryo, *H. crassispina* observed by scanning electron microscopy. Scale bars: 1 μm.

Movie 5. A basal body, rootlet, and a giant Golgi apparatus in *H. crassispina*. A movie made from 30 nm-thick sections of a giant Golgi apparatus, basal body, and its rootlet in the cells of the sea urchin embryo *H. crassispina* observed by scanning electron microscopy. Scale bars: 1 μm.

Movie 6. Clustered Golgi stacks in *Acanthosepion esculentum*. A movie made from 90 nm-thick 15 sections of a cell in the mollusk *A. esculentum* embryo, was observed by scanning electron microscopy. Scale bars: 5 μm (the first half) and 1 μm (the latter half).
