# Supplementary material for: Cell-wide arrangement of Golgi/RE units depends on the microtubule organization
Source: Cell Struct Funct. 2024 Oct 3;49(2):101–10. doi: 10.1247/csf.24055 (PMC11930777; doi:10.1247/csf.24055)
Supplement: Supplementary file 7 — Supplementary Materials [file csf_49_24055_7.pdf]

Supplemental information

Movie 1. A giant Golgi apparatus under the microtubule array

Projection images made from 31 sections with 0.3-μm intervals in the embryo cells of the sea urchin *Hemicentrotus pulcherrimu*. *Trans*-Golgi cisternae and microtubules were visualized using GalT::EGFP (magenta) after the mRNA was injected into fertilized oocytes. The samples were immunostained with anti-α-tubulin antibody (green) at late blastula stage.

Scale bars: 5 μm.

Movie 2. Side view of a giant Golgi apparatus and RE under the microtubule array and cilium

A movie made from 55 sections with 0.3-μm intervals in the embryo cells of the sea urchin *H. pulcherrimu*. *Trans*-Golgi cisternae, REs, and microtubules were visualized using GalT::EGFP (green) and tdTomato::Vamp3 (red) after injecting their mRNA into fertilized oocytes. The samples were immunostained with anti-α-tubulin antibody (blue) at the late blastula stage.

Scale bars: 5 μm.

Movie 3. A giant Golgi apparatus and RE under the microtubule array and cilium

A movie made from 23 sections with 0.3-μm intervals in the embryo cells of the sea urchin *H. pulcherrimu*. *Trans*-Golgi cisternae, REs, and microtubules were visualized using GalT::EGFP (green) and tdTomato::Vamp3 (red) after injecting their mRNA into fertilized oocytes. The samples were immunostained with anti-α-tubulin antibody (blue) at the late blastula stage.

Scale bars: 5 μm.

Movie 4. Rootlet of a cilium wrapped by a giant Golgi apparatus in *Heliocidaris crassispina*

A movie was made from 30 nm-thick 28 sections of a giant Golgi apparatus (yellow), basal body (blue), cilium (blue), and its rootlet (red) in the cells of the sea urchin embryo, *H. crassispina* observed by scanning electron microscopy.

Scale bars: 1 µm.

Movie 5. A basal body, rootlet, and a giant Golgi apparatus in *H. crassispina*

A movie made from 30 nm-thick sections of a giant Golgi apparatus, basal body, and its rootlet in the cells of the sea urchin embryo *H. crassispina* observed by scanning electron microscopy.

Scale bars: 1 µm.

Movie 6. Clustered Golgi stacks in *Acanthosepion esculentum*

A movie made from 90 nm-thick 15 sections of a cell in the mollusk *A. esculentum* embryo, was observed by scanning electron microscopy.

Scale bars: 5 µm (the first half) and 1 µm (the latter half).
